# Atypical Lipomatous Tumor/Well-Differentiated Liposarcoma Developed in a Patient with Progressive Muscular Dystrophy: A Case Report and Review of the Literature

**DOI:** 10.1155/2017/3025084

**Published:** 2017-05-29

**Authors:** Ryo Miyagi, Toshihiko Nishisho, Shinjiro Takata, Yoshimitsu Shimatani, Shunichi Toki, Koichi Sairyo

**Affiliations:** ^1^Department of Orthopedics, Institute of Biomedical Sciences, Tokushima University Graduate School, Tokushima 770-8503, Japan; ^2^Department of Clinical Neuroscience, Institute of Biomedical Sciences, Tokushima University Graduate School, Tokushima, Japan; ^3^Department of Orthopedics, National Hospital Organization Tokushima Hospital, Tokushima, Japan

## Abstract

**Background:**

Atypical lipomatous tumor/well-differentiated liposarcoma (ALT/WDLS) is an intermediate or locally aggressive form of adipocytic soft tissue sarcoma. Muscular dystrophy (MD) is characterized by progressive muscle atrophy and its replacement by adipose and fibrous tissue. Recently, some authors have reported that MD genes are related to neoplastic formation, but there have been no detailed clinical reports of ALT associated with MD.

**Case Presentation:**

A 73-year-old woman with a diagnosis of limb-girdle MD visited our department for recurrence of a huge tumor in her left thigh. She had undergone resection of a lipoma at the same site more than 20 years earlier. Imaging studies revealed a lipomatous tumor in her left thigh. We performed marginal resection including the adjacent muscles. Histological diagnosis was atypical lipomatous tumor. The postoperative course was uneventful, with no recurrence at 36 months of follow-up.

**Conclusion:**

We encountered a huge atypical tumor in a patient with MD. This is the first detailed report to describe an association between ALT and MD. We hypothesize that degenerative changes occurring in adipose tissue during muscle atrophy can cause lipomatous neoplasms and moreover that the mutation of MD-related genes may lead to the proliferation of tumor cells or to malignancy.

## 1. Background

Limb-girdle muscular dystrophy (LGMD) is characterized by progressive weakness of proximal muscles, of which one type involves the replacement of muscle by adipose and fibrous tissue [1–6]. Most muscular dystrophies (MDs) are caused by mutations in genes such as dystrophin, dysferlin, calpain, or LARGE like-glycosyltransferase [7–12].

Recently, these genes have been shown to be strongly related to neoplastic lesions. Schmidt et al. suggested that these genes might serve as tumor suppressors [12]. Several types of tumor, such as myeloma and rhabdomyosarcoma, have been reported to be related to MDs [13, 14]. Here, we report the case of a 73-year-old patient with LGMD type 2B (LGMD2B) who developed an atypical lipomatous tumor (ALT), an intermediate or locally aggressive form of adipocytic tumor that is also referred to as well-differentiated liposarcoma (WDLS) [15].

## 2. Case Presentation

A 73-year-old woman with progressive weakness in the proximal muscles since age 50 was diagnosed with LGMD. Her parents were first cousins, and of her four younger siblings, three were diagnosed with LGMD. The patient underwent resection of a soft tissue tumor in the left thigh at age 49. The pathological diagnosis was lipoma.

**Figure 1:**
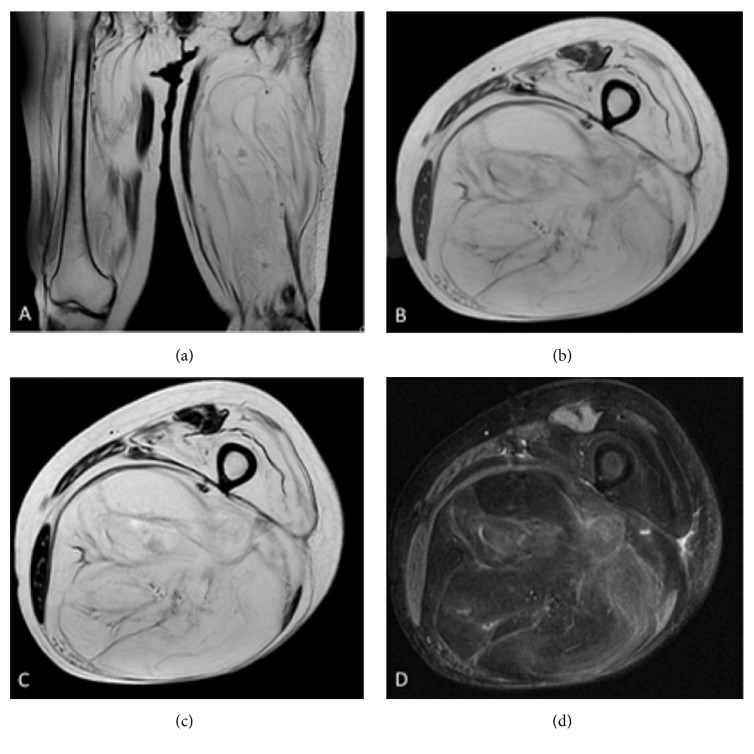
Magnetic resonance imaging revealed a tumor mass measuring 13 × 15 × 24 cm located in the left posterior thigh. Almost all the muscles, except the rectus femoris, sartorius, and gracilis muscles, show lipomatous changes. T1- and T2-weighted images (WIs) T2-WI, and short-TI inversion recovery (STIR) images show high intensity. Coronal T2-WI (a), T1-WI (axial view) (b), T2-WI (axial view) (c), and STIR images (axial view) (d).

The tumor recurred in the left thigh at age 62. At the time of presentation to our department, the tumor had been present for 11 years and had gradually increased in size. Physical examination at first visit revealed an elastic hard mass measuring approximately 25 cm in width and 30 cm in length, located in the posterior left thigh. No tenderness, redness, or local warmth was noted. Laboratory examinations revealed that serum creatine kinase (CK) was mildly elevated at 825 *μ*IU/L. Plain radiography showed a soft tissue mass shadow in the left thigh without calcification or periosteal reaction. Magnetic resonance imaging (MRI) revealed high signal intensity on T1- and T2-weighted images and heterogeneous hyperintensity on short-TI inversion recovery images (Figure 1). In addition, severe muscle atrophy and lipomatous changes were observed in both thighs. Open biopsy revealed no evidence of highly malignant cells. Therefore, we considered the tumor to be a lipoma or an atypical lipomatous tumor.

**Figure 2:**
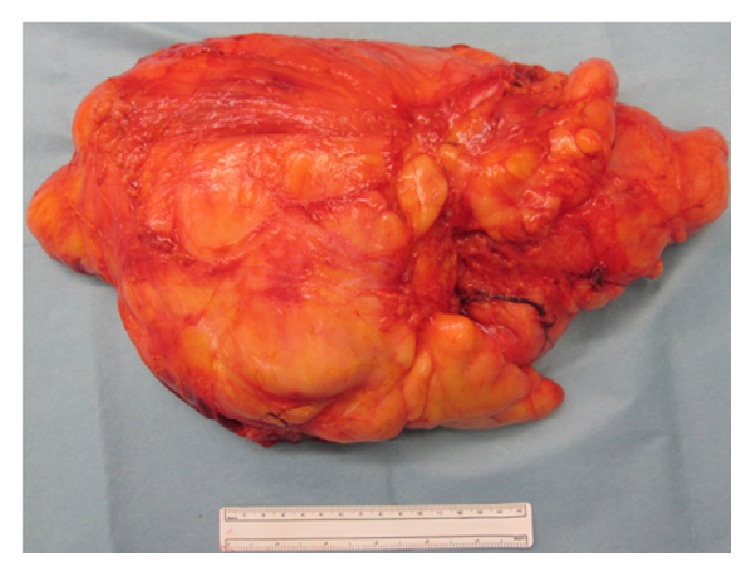
Macroscopic appearance of the resected tumor. The mass includes the semitendinosus muscle, semimembranosus muscle, and biceps femoris muscle.

**Figure 3:**
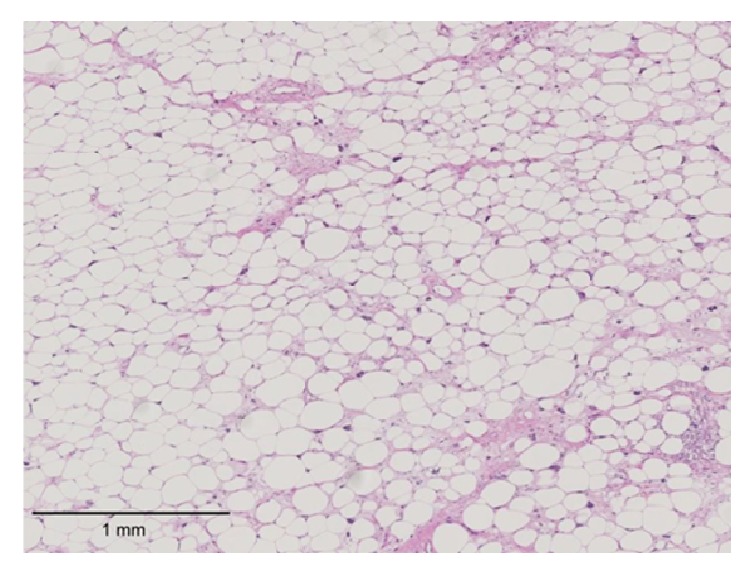
Pathological findings show mature adipocytes with cellular atypia and marked variations in size and shape. Hematoxylin-eosin staining, ×25. Scale bar, 1 mm.

Surgical excision of the tumor was performed. On exploration, the tumor consisted of fat globules partially surrounded by a capsule and with poorly defined margins that was infiltrating different layers of the thigh muscles. We performed marginal resection including the semitendinosus muscle, semimembranosus muscle, and biceps femoris muscle (Figure 2). The resected tumor measured 15 × 35 × 20 cm. Pathological examination revealed that it contained mature adipocytes with cellular atypia (Figure 3). Immunohistochemical expression was positive for p16, MDM2, and CDK4. The final pathological diagnosis was atypical lipomatous tumor. The patient's postoperative course was uneventful, and there was no evidence of recurrence at 36 months postoperatively.

Since western blot analysis of muscle showed a deficit of dysferlin, the patient was finally diagnosed with LGMD2B.

## 3. Discussion

LGMD is characterized by progressive weakness of proximal muscles such as those of the hip or shoulder and was first proposed as a nosological entity by Walton and Nattrass in 1954 [1]. LGMD is classified into two main groups: autosomal dominant (LGMD type 1) and autosomal recessive (LGMD type 2). Autosomal recessive inheritance is more common. The symptoms of LGMD usually occur in the first and second decades of life; however, the onset, progression, and distribution of the weakness and wasting differ among the disease subtypes [1–6]. Our patient noticed muscle atrophy at age 50. Several LGMD subtypes have recently been identified based upon mutations of certain genes [7–12]; for example, mutations in the calpain 3 and dysferlin genes cause LGMD2A and LGMD2B, respectively.

Recently, some authors have indicated an association between MD and neoplasms [12, 14, 16–20]. MD genes including dystrophin, dysferlin, and calpain 3 act as tumor suppressor genes [21]. Hosur et al. reported that a dystrophin and dysferlin double mutant mice model of human Duchenne Muscular Dystrophy and of LGMD2B each developed rhabdomyosarcoma at an average age of 12 months, with an incidence of >90% [14]. Schmidt et al. showed that mutations in MD genes (Dmd, Dysf, Capn3, and LARGE) lead to the spontaneous formation of skeletal muscle-derived malignant tumors in mice, presenting as mixed rhabdomyo-, fibro-, and liposarcomas [12]. The observations of Schmidt et al. suggest that genetically distinct MDs in humans and mice may share a common pathology of cancer-like DNA damage and genomic instability [12].

Liposarcoma is the most common soft tissue sarcoma and accounts for approximately 20% of all mesenchymal malignancies encountered by practicing surgical pathologists [15]. According to the WHO Classification of Tumors of Soft Tissue and Bone 2013, liposarcomas mainly have four histological subtypes: ALT/WDLS, dedifferentiated liposarcoma, myxoid liposarcoma, and pleomorphic liposarcoma. ALT/WDLS is low to intermediate grade tumors/malignancy and dedifferentiated liposarcoma, myxoid round cell liposarcoma, and pleomorphic liposarcoma are high grade tumors/malignancy. The concept of ALT/WDLS was first reported by Evans et al. in 1979 [22]. They reported that WDLS of subcutaneous tissue and the extremities showed no metastasis, and recommended that the term “well-differentiated liposarcoma” be retained for cases of retroperitoneal tumors because it was difficult in those cases to achieve complete resection and the patients died of local recurrence.

To obtain a definitive diagnosis of ALT based on imaging findings is sometimes difficult because the tumor resembles lipoma. Using standard imaging characteristics (i.e., large size, thick septa, nodules, and nonfatty areas) provides high sensitivity but poor specificity for the diagnosis of ALT due to overdiagnosis of ALT/WDLS from imaging findings [23].

Histologically, ALT is formed by the proliferation of relatively mature adipocytes, consisting of a mixture of mature adipocytes and fibrous connective tissue. The immunohistochemical expression of p16, MDM2, and CDK4 is useful for distinguishing ALT from other lipomatous tumors [24]. In the present case, expression was positive for p16, MDM2, and CDK4, and a definitive diagnosis was thus obtained.

Few reports have examined an association between lipomatous tumors and MD. Yagi et al. reported the association between lipoma and myotonic dystrophy in 2011 [16]. We hypothesize that degenerative changes occurring in adipose tissue during muscle atrophy may cause lipomatous neoplasms; moreover, the mutation of MD-related genes, such as dysferlin, may lead to the proliferation of the tumor or turn into a malignant tumor. To date, to the best of our knowledge, this is the first detailed clinical report to describe an association between ALT and MD, specifically with LGMD2B.

## Abbreviations

ALT: Atypical lipomatous tumor
WDLS: Well-differentiated liposarcoma
LGMD: Limb-girdle muscular dystrophy.

## Additional Points

*Availability of Data and Materials.* The datasets supporting the conclusions of this article are included within the article.
